# Supplementary material for: Floquet group theory and its application to selection rules in harmonic generation
Source: Nat Commun. 2019 Jan 24;10:405. doi: 10.1038/s41467-018-07935-y (PMC6345759; doi:10.1038/s41467-018-07935-y)
Supplement: Supplementary file 1 — Supplementary Information [file 41467_2018_7935_MOESM1_ESM.pdf]

# Supplementary information
# Floquet group theory and its application to selection rules in harmonic generation

*Ofer Neufeld[1,2], Daniel Podolsky[2] and Oren Cohen[1,2]*
[1]Solid state Institute, Technion - Israel Institute of Technology, Haifa 32000, Israel.
[2]Physics Department, Technion - Israel Institute of Technology, Haifa 32000, Israel.

The supplementary information file contains the following: in supplementary note 1 we show that the selection rules arising from dynamical symmetries (DSs) can be treated as eigenvalue problems in the frequency-domain. In supplementary note 2 we prove that the generating operators of a dynamical group are sufficient to describe the selection rules in Floquet systems. Supplementary note 3 derives the selection rules for harmonic generation (HG) spectra and outlines the connection to perturbative derivations. Supplementary note 4 provides a list of selected dynamical groups as developed in the main text. Supplementary note 5 discusses similarities between Floquet and other groups. Lastly, supplementary notes 6-8, present numerical time dependent Schrödinger equation (TDSE) HG calculations for novel DSs (both in (2+1)D and (3+1)D), from atomic gas, oriented and non-oriented molecular gas, and solids, demonstrating that the analytically derived selection rules are upheld, and exploring DS breaking spectroscopy.

## Supplementary note 1: DS constraints as eigenvalue problems

We show here how each DS constraint on a physical observable (derived in Eq. (22) in the main text) can be written as a set of eigenvalue problems in the Fourier domain. In order to understand how this takes place, we first demonstrate it for a general observable $\mathbf{o}(t)$ in a (2+1)D system with $\hat{C}_2 = \hat{\tau}_2 \cdot \hat{R}_2$ DS. Since $\mathbf{o}(t)$ is $T$-periodic ($T$-periodicity is a symmetry of all Floquet systems), it can be expanded to a Fourier series:

$$\mathbf{o}(\mathrm{t}) = \sum_n \mathbf{F}_\mathrm{n} \exp\left(in\frac{2\pi t}{T}\right) \qquad (1)$$

where the coefficients $\mathbf{F}_\mathrm{n}$ are complex numbers. Thus, the Fourier transform of $\vec{o}(t)$ is discrete in the frequency domain, with Dirac delta functions around integer multiples of the frequency $\omega_0 = 2\pi/T$. Furthermore, if $\mathbf{o}(t)$ is a real quantity, then, the coefficients real parts are even, and their imaginary parts are odd:

$$\mathbf{F}_\mathrm{n} = \mathbf{a}_\mathrm{n} + i\mathbf{b}_\mathrm{n}; \mathbf{a}_\mathrm{n} = \mathbf{a}_{-\mathrm{n}}, \mathbf{b}_\mathrm{n} = -\mathbf{b}_{-\mathrm{n}} \qquad (2)$$

where $\mathbf{a}_\mathrm{n}$ and $\mathbf{b}_\mathrm{n}$ are real. Applying the symmetry constraint on supplementary Eq. (1), that is, requiring that the observable is invariant to $\hat{C}_2$, yields:

$$\sum_n \hat{C}_2 \cdot \mathbf{F}_\mathrm{n} \exp\left(in\frac{2\pi t}{T}\right) = \sum_n \mathbf{F}_\mathrm{n} \exp\left(in\frac{2\pi t}{T}\right) \qquad (3)$$

The coefficients of identical exponents in supplementary Eq. (3) must be equal. This reduces supplementary Eq. (3) to separate eigenvalue problems for each frequency component $n$:

$$\hat{R}_2 \cdot \mathbf{F}_\mathrm{n} = e^{in\pi}\mathbf{F}_\mathrm{n} \qquad (4)$$

The rotational operation $\hat{R}_2$ has two identical eigenvalues of value -1, with any eigenvector. The constraints in supplementary Eq. (4) are upheld if the temporal part of the symmetry (that translates by half a period) leads to an eigenvalue that corresponds to those of the matrix $\hat{R}_2$, which only happens for odd values of $n$. That is, the equation is solved by any eigenvector for odd values of $n$, and has only a trivial solution for even values of $n$. All DSs can be treated in a similar manner, while only the eigenvalues and eigenvectors that uphold the equations change for different DSs. These constraints restrict the spectrum of the physical observable, and therefore its temporal evolution as well. A solution is always guaranteed: either a non-trivial one, or a trivial one ($\mathbf{F}_\mathrm{n} = 0$). For HG, a trivial solution results in "forbidden harmonics", that is, selection rules. More generally, selection rules can be expressed as any relation between the components of $\mathbf{F}_\mathrm{n}$, determined by the eigenvectors themselves. A similar derivation for a $\hat{C}_3$ symmetry which is present in counter-rotating bi-circular $\omega$-2$\omega$ laser fields, was presented in ref. 1.

## Supplementary note 2: Selection rules in groups with multiple members

In this note we prove that the generators of a symmetry group are sufficient to describe the selection rules and other requirements in Floquet systems. This is intuitive, since the generators provide all the information about the group (other members in the group result in restrictions that are upheld by closure and associativity in the group). First, we consider a group $G$ with a single generator, $\hat{X}$. The generator can be broken to spatial and temporal components:

$$\hat{X} = \hat{X}_{temp} \cdot \hat{X}_{space} \tag{5}$$

The resulting eigenvalue problems that represent the symmetry constraints due to $\hat{X}$ can be written as:

$$\sum_n \hat{X}_{space} \cdot \mathbf{F}_\mathrm{n} \cdot \hat{X}_{temp} \cdot \exp\left(in\frac{2\pi t}{T}\right) = \sum_n \mathbf{F}_\mathrm{n} \cdot \exp\left(in\frac{2\pi t}{T}\right) \tag{6}$$

The temporal part determines the eigenvalues, and the spatial part determines the operator to be diagonalized. A non-trivial solution exists only if the spatial and the temporal parts exactly match. Assuming a solution $\mathbf{F}_\mathrm{n}$ that solves supplementary Eq. (6), symmetry operations that are powers of $\hat{X}$ lead to eigenvalue problems with the same solutions. The $m$'th power leads to:

$$\sum_n \left(\hat{X}_{space}\right)^m \cdot \mathbf{F}_\mathrm{n} \cdot \left(\hat{X}_{temp}\right)^m \cdot \exp\left(in\frac{2\pi t}{T}\right) = \sum_n \mathbf{F}_\mathrm{n} \cdot \exp\left(in\frac{2\pi t}{T}\right) \tag{7}$$

where we used the commutativity of the spatial and temporal operators. Due to associativity in the group, the equation above is solved by the same $\mathbf{F}_\mathrm{n}$, with the eigenvalues of $\hat{X}$ to the power of $m$. Thus, symmetry constraints resulting from powers of $\hat{X}$ coincide with those imposed by $\hat{X}$.

Next, we consider two generators, $\hat{X}$ and $\hat{Y}$ and their resulting symmetry constraints. Again, we assume a solution $\mathbf{F}_\mathrm{n}$ that simultaneously solves the eigenvalue problems of $\hat{X}$ and $\hat{Y}$ combined. In this case, the same solution also solves the eigenvalue problem defined by the symmetry operation that is $\hat{X} \cdot \hat{Y}$:

$$\sum_n \left(\hat{X}_{space} \cdot \hat{Y}_{space}\right) \cdot \mathbf{F}_\mathrm{n} \cdot \left(\hat{X}_{temp} \cdot \hat{Y}_{temp}\right) \cdot \exp\left(in\frac{2\pi t}{T}\right) = \sum_n \mathbf{F}_\mathrm{n} \cdot \exp\left(in\frac{2\pi t}{T}\right) \tag{8}$$

where we again used the commutativity of the spatial and temporal operators. Supplementary Eq. (8) can be thought of as a series multiplication, or as a single eigenvalue problem whose eigenvalues are multiplications of those of $\hat{X}$ and $\hat{Y}$. Clearly, if all symmetry constraints due to powers of $\hat{X}$ and $\hat{Y}$ and their products are upheld, then the same solution is valid for all symmetry constraints in the group, since the group is comprised of powers and products of its generators. At this point the theorem is trivially extended to any number of generators, and results in the following: given a dynamical group $G$, one must only examine the selection rules arising from the generating symmetries.

## Supplementary note 3: HG selection rules

We develop the HG selection rules for underlying DSs using the eigenvalue problem approach described in supplementary note 1 for the time-dependent polarization (Eq. (24) in the main text), which is even under time-reversal (odd observables, e.g. currents, have slightly different selection rules). We begin with the (2+1)D case that describes DSs in collinear atomic/molecular HG, and then move on to the (3+1)D case that describes non-collinear and solid HG.

### HG selection rules in (2+1)D

### Time reversal symmetry - $\hat{\mathbf{T}}$

$\hat{\mathrm{T}}$ symmetry results in the following constraints:

$$\mathbf{F}_\mathrm{n} = \mathbf{F}_{-\mathrm{n}} \tag{9}$$

A general solution demands the $x$ and $y$ components of $\mathbf{F}_\mathrm{n}$ are in phase, meaning any driver invariant under time-reversal results in linearly-polarized only harmonics.

### Reflection-time reversal symmetry - $\hat{\boldsymbol{D}}_{\boldsymbol{x}}$

$\hat{D}_x$ symmetry results in the following constraints:

$$\hat{\sigma}_x \cdot \mathbf{F}_\mathrm{n} = \mathbf{F}_{-\mathrm{n}} \tag{10}$$

The selection rules dictate that the $x$ and $y$ components have a relative phase of π/2. This means any elliptical emission is allowed, as long as the polarization ellipse has a major/minor axis parallel to the reflection axis.

**Reflection-time translation symmetry - $\hat{\boldsymbol{Z}}_{\boldsymbol{x}}$**

$\hat{Z}_x$ symmetry results in the following constraints:

$$\hat{\sigma}_x \cdot \mathbf{F}_\mathrm{n} = e^{in\pi}\mathbf{F}_\mathrm{n} \tag{11}$$

Here we must separate to even and odd $n$ cases. The general solution dictates selection rules where even harmonics are linearly-polarized along the symmetry axis, and odd harmonics are linearly-polarized orthogonally to the symmetry axis. This selection rule was demonstrated experimentally for a cross-linear ω-2ω pumps driving HHG in refs. 2,3.

**Reflection-time translation followed by time reversal symmetry - $\hat{\boldsymbol{H}}_{\boldsymbol{x}}$**

$\hat{H}_x$ symmetry results in the following constraints:

$$\hat{\sigma}_x \cdot \mathbf{F}_\mathrm{n} = e^{in\pi}\mathbf{F}_{-\mathrm{n}} \tag{12}$$

The resulting selection rules dictate that the $x$ and $y$ components have a relative phase of π/2. That is, any elliptical emission is allowed, as long as the polarization ellipse has a major/minor axis parallel to the reflection axis. The identical selection rules to the operator $\hat{D}_x$ are not coincidental, and indicate that it does not matter which point in time upholds $\hat{D}_x$ symmetry (the additional time-translation operator shifts the $\hat{S}_x$ symmetry by $T/2$).

**Rotation by 180⁰-time translation symmetry - $\hat{\boldsymbol{C}}_{\boldsymbol{2}}$**

$\hat{C}_2$ symmetry was previously found responsible for odd-only harmonic emission from one-color laser fields[4], as we also show here. $\hat{C}_2$ symmetry results in the following constraints:

$$\hat{R}_2 \cdot \mathbf{F}_\mathrm{n} = e^{in\pi}\mathbf{F}_\mathrm{n} \tag{13}$$

The spatial rotation operator by 180° degrees has two identical eigenvalues of value -1. A non-trivial solution then only exists for odd values of $n$. This results in odd-only harmonics, compatible with previous derivations, with no particular limitation on the polarization. For instance, slightly elliptical fields uphold this symmetry and produce elliptically polarized odd harmonics[5,6].

**Rotation by 180⁰-time reversal symmetry - $\hat{\boldsymbol{Q}}$**

$\hat{Q}$ symmetry results in the following constraints:

$$\hat{R}_2 \cdot \mathbf{F}_\mathrm{n} = \mathbf{F}_{-\mathrm{n}} \tag{14}$$

The resulting selection rules dictate that the $x$ and $y$ components of $\mathbf{F}_\mathrm{n}$ are in phase. Thus, any system upholding $\hat{Q}$ symmetry generates only linearly-polarized harmonics.

**Rotation by 180⁰-time translation followed by time reversal symmetry - $\hat{\boldsymbol{G}}$**

$\hat{G}$ symmetry results in the following constraints:

$$\hat{R}_2 \cdot \mathbf{F}_\mathrm{n} = e^{in\pi}\mathbf{F}_{-\mathrm{n}} \tag{15}$$

For which the resulting selection rules are identical to those imposed by $\hat{Q}$ symmetry, with similar arguments.

**Rotation-time translation symmetry - $\hat{\boldsymbol{C}}_{\boldsymbol{N}}$**

$\hat{C}_N$ symmetry results in the following constraints:

$$\hat{R}_N \cdot \mathbf{F}_\mathrm{n} = e^{in\frac{2\pi}{N}}\mathbf{F}_\mathrm{n} \tag{16}$$

The rotational operator has two eigenvalues: $e^{\pm i2\pi/N}$, with the eigenvectors: $\begin{pmatrix}1\\ \pm i\end{pmatrix}$. The selection rules dictate only circularly polarized $n = Nq \pm 1$ harmonics are emitted (for integer $q$), with alternating helicities. This derivation appeared in ref. 7.

**Generalized rotation-time translation symmetry - $\hat{\boldsymbol{C}}_{\boldsymbol{N,m}}$**

This symmetry is exhibited in HHG driven by generalized two-color bi-circular EM fields[8,9]. $\hat{C}_{N,m}$ symmetry results in the following constraints:

$$\hat{R}_{N,m} \cdot \mathbf{F}_\mathrm{n} = e^{in\frac{2\pi}{N}}\mathbf{F}_\mathrm{n} \tag{17}$$

The resulting selection rules dictate only circularly polarized $n = Nq \pm m$ harmonics are emitted (for integer $q$), with alternating helicities, consistent with previous derivations from conservation laws[8,9].

**Generalized elliptical rotation-time translation symmetry - $\hat{e}_{N,m}$**

$\hat{e}_{N,m}$ symmetry results in the following constraints:

$$\hat{L}_b \cdot \hat{R}_{N,m} \cdot \hat{L}_{1/b} \cdot \mathbf{F}_\mathrm{n} = e^{in\frac{2\pi}{N}}\mathbf{F}_\mathrm{n} \qquad (18)$$

The generalized rotation-scaling operator has the same eigenvalues as the standard rotational operator, but different eigenvectors that depend on the ellipticity: $\begin{pmatrix} 1 \\ \pm bi \end{pmatrix}$. The resulting selection rules dictate that the emitted harmonics are elliptically polarized along the elliptical axis with alternating helicity, with the same underlying ellipticity $b$, and only $n = Nq \pm m$ harmonics are emitted (for integer $q$).

**HG Selection rules in (3+1)D**

The (3+1)D formulation is identical to the (2+1)D case, except that rotations must now revolve around a specific axis in 3D space (which we always assume is the z-axis), and reflections are no longer reflection axes, but reflection planes (which we always assume to be the xy plane). Additionally, $\mathbf{F}_\mathrm{n}$ is now a 3D object.

**Time reversal symmetry - $\hat{\mathrm{T}}$**

$\hat{\mathrm{T}}$ symmetry results in the following constraints:

$$\mathbf{F}_\mathrm{n} = \mathbf{F}_{-\mathrm{n}} \qquad (19)$$

A general solution demands the $x$, $y$, and $z$ components of $\mathbf{F}_\mathrm{n}$ are in phase, meaning any driver invariant under time-reversal results in linearly-polarized only harmonics, same as the (2+1)D case.

**Reflection-time reversal symmetry - $\hat{D}_{xy}$**

$\hat{D}_{xy}$ symmetry results in the following constraints:

$$\hat{\sigma}_{xy} \cdot \mathbf{F}_\mathrm{n} = \mathbf{F}_{-\mathrm{n}} \qquad (20)$$

The selection rules dictate that the $x$ and $y$ components are in phase (linear polarization within the $xy$ plane), and are phase shifted by $\pi/2$ for the $z$-axis induced polarization. This means the 3D polarization ellipsoid has a 2D projection for each harmonic with a major/minor axis along the $z$-axis.

**Reflection-time translation symmetry - $\hat{Z}_{xy}$**

$\hat{Z}_{xy}$ symmetry results in the following constraints:

$$\hat{\sigma}_{xy} \cdot \mathbf{F}_\mathrm{n} = e^{in\pi}\mathbf{F}_\mathrm{n} \qquad (21)$$

Here we must separate to even and odd $n$ cases. The general solution dictates selection rules where even harmonics are in any elliptical polarization within the symmetry plane, and odd harmonics must be linearly-polarized orthogonally to the symmetry plane.

**Reflection-time translation followed by time reversal symmetry - $\hat{H}_{xy}$**

$\hat{H}_{xy}$ symmetry results in the following constraints:

$$\hat{\sigma}_{xy} \cdot \mathbf{F}_\mathrm{n} = e^{in\pi}\mathbf{F}_{-\mathrm{n}} \qquad (22)$$

The resulting selection rules are identical to those of the operator $\hat{D}_x$.

**Rotation by 180$^0$-time translation symmetry - $\hat{C}_2$**

$\hat{C}_2$ symmetry results in the following constraints:

$$\hat{R}^z{}_2 \cdot \mathbf{F}_\mathrm{n} = e^{in\pi}\mathbf{F}_\mathrm{n} \qquad (23)$$

The selection rules lead to odd-only harmonics that can be polarized within the plane orthogonal to the rotation ($xy$ plane), and only even harmonics polarized parallel to the symmetry axis ($z$-axis).

**Rotation by 180$^0$-time reversal symmetry - $\hat{Q}$**

$\hat{Q}$ symmetry results in the following constraints:

$$\hat{R}_2 \cdot \mathbf{F}_\mathrm{n} = \mathbf{F}_{-\mathrm{n}} \qquad (24)$$

The resulting selection rules dictate that the $x$ and $y$ polarization components are in phase (linear polarization within the $xy$ plane), and phase shifted by $\pi/2$ from the $z$ symmetry axis. Thus, the 3D polarization ellipsoid has a 2D projection for each harmonic with a major/minor axis along the $z$-axis.

**Rotation by 180$^0$-time translation followed by time reversal symmetry - $\hat{G}$**

$\hat{G}$ symmetry results in the following constraints:

$$\hat{R}_2 \cdot \mathbf{F}_\mathrm{n} = e^{in\pi}\mathbf{F}_{-\mathrm{n}} \quad (25)$$

For which the resulting selection rules are identical to those imposed by $\hat{Q}$ symmetry.

**Inversion-time translation symmetry - $\hat{\boldsymbol{F}}$**

$\hat{F}$ symmetry results in the following constraints:

$$\hat{\imath} \cdot \mathbf{F}_\mathrm{n} = e^{in\pi}\mathbf{F}_\mathrm{n} \quad (26)$$

The selection rules lead to odd-only harmonics with any generalized polarization. We note this symmetry is well-known from perturbative nonlinear optics, and often attributed to centro-symmetric media which cancel out even order nonlinear coefficients[10,11]. In our case, $\hat{F}$ symmetry is the generalization of this selection rule, which also depends on the DS of the incident pump field.

**Inversion-time reversal symmetry - $\hat{\boldsymbol{J}}$**

$\hat{J}$ symmetry results in the following constraints:

$$\hat{\imath} \cdot \mathbf{F}_\mathrm{n} = \mathbf{F}_{-\mathrm{n}} \quad (27)$$

The resulting selection rules dictate that the $x$, $y$ and $z$ axes induced polarization components are in phase (any linearly polarized high harmonic emission is allowed).

**Inversion-time translation followed by time reversal symmetry - $\hat{\boldsymbol{A}}$**

$\hat{A}$ symmetry results in the following constraints:

$$\hat{\imath} \cdot \mathbf{F}_\mathrm{n} = e^{in\pi}\mathbf{F}_{-\mathrm{n}} \quad (28)$$

For which the resulting selection rules are identical to those imposed by $\hat{J}$ symmetry.

**Generalized elliptical rotation-time translation symmetry - $\hat{\boldsymbol{e}}_{\boldsymbol{N,m}}$**

$\hat{e}_{N,m}$ symmetry results in the following constraints:

$$\hat{L}^y{}_b \cdot \hat{R}^z{}_{N,m} \cdot \hat{L}^y{}_{1/b} \cdot \mathbf{F}_\mathrm{n} = e^{in\frac{2\pi}{N}}\mathbf{F}_\mathrm{n} \quad (29)$$

The resulting selection rules dictate that the emission of $n = Nq \pm m$ (for integer $q$) harmonics is permitted with elliptical polarization and alternating helicity along the elliptical axis and within the plane orthogonal to the rotation axis, with the same underlying ellipticity $b$. The emission of $n = Nq$ harmonics is also permitted, but only with linear polarization parallel to the symmetry axis. This operation reduces to circular symmetry ($\hat{C}_{N,m}$) for $b = 1$.

**Elliptical improper rotation-time translation symmetry – even orders - $\hat{\boldsymbol{P}}_{\boldsymbol{2N,m}}$**

$\hat{P}_{2N,m}$ symmetry results in the following constraints:

$$\left[\hat{\sigma}_{xy} \cdot \left(\hat{L}^y{}_b \cdot \hat{R}^z{}_{2N,m} \cdot \hat{L}^y{}_{1/b}\right)\right] \cdot \mathbf{F}_\mathrm{n} = e^{in\frac{\pi}{N}}\mathbf{F}_\mathrm{n} \quad (30)$$

The resulting selection rules dictate that the emission of $n = 2Nq \pm m$ (for integer $q$) harmonics is permitted with elliptical polarization and alternating helicity along the elliptical axis and within the plane orthogonal to the rotation axis, with the same underlying ellipticity $b$. The emission of $n = N(2q + 1)$ harmonics is also permitted, but only with linear polarization parallel to the symmetry axis. This operation reduces to circular improper rotational symmetry ($\hat{M}_{2N,m}$) for $b = 1$.

**Elliptical improper rotation-time translation symmetry – odd orders - $\hat{\boldsymbol{P}}_{\boldsymbol{2N+1,m}}$**

$\hat{P}_{2N+1,m}$ symmetry results in the following constraints:

$$\left[\hat{\sigma}_{xy} \cdot \left(\hat{L}^y{}_b \cdot \hat{R}^z{}_{2N+1,m} \cdot \hat{L}^y{}_{1/b}\right)\right] \cdot \mathbf{F}_\mathrm{n} = e^{in\frac{\pi}{2N+1}}\mathbf{F}_\mathrm{n} \quad (31)$$

The resulting selection rules dictate that the emission of $n = 2q(2N + 1) \pm m$ (for integer $q$) harmonics is permitted with elliptical polarization and alternating helicity along the elliptical axis, and within the plane orthogonal to the rotation axis, with the same underlying ellipticity $b$. The emission of $n = (2N + 1)(2q + 1)$ harmonics is also permitted, but only with linear polarization parallel to the symmetry's rotation axis. This operation reduces to circular improper rotational symmetry ($\hat{M}_{2N+1,m}$) for $b = 1$.

**Connection between DS group theory and perturbative selection rules**

In this subsection we directly outline the connections between our general DS-based selection rules derivation for HG, and the standard perturbative derivation of selection rules in non-linear optics (NLO). We also discuss the connection between the medium's static symmetries and the selection rules presented in the main text.

First, we comment on the role of the medium's characteristic point-group symmetry (describing the static symmetries of the field-free system, either gas, liquid, or solid) in the DS-based theory. In practice, the combination of the crystal/molecule symmetry and pump symmetry is done by taking the following steps:

(i) Identify the symmetry of the medium using standard tools (molecular group theory for molecules, and crystallographic space group theory for solids).
(ii) Identify the DSs of the pump.
(iii) Identify the DSs of the joint system (pump and medium), that is, the static symmetries of the medium that are also elements in the DSs of the pump.
(iv) Find the selection rules corresponding to the DSs of the joint system in the tables presented in the main text.

This approach is general, and can be applied to any target with one of the 32 known point groups systematically according to the symmetry element. As an example, consider an α-$Al_2O_3$ single crystal interacting with an $\omega$-$2\omega$ bi-circular pump[12] propagating along the *c*-axis of the crystal. In this case, we identify α-$Al_2O_3$ belonging to the R$\overline{3}$c space group that exhibits 3-fold symmetry ($\hat{R}_3$) along the $c$-axis[13]. Similarly, we identify the bi-circular pump's dynamical symmetry group that exhibits $\hat{C}_3$ DS, which have an identical $\hat{R}_3$ spatial part. Hence, $\hat{C}_3$ is a joint DS for the light-matter system, leading to HG selection rules according to Table 1 in the main text. On the other hand, a single-crystal of rocksalt MgO belongs to the space group Fm$\overline{3}$m that does not exhibit $\hat{R}_3$ symmetry; hence, if MgO is pumped by the same bi-circular beam, $\hat{C}_3$ is not a joint DS of the light-matter system, and does not lead to selection rules.

Second, we comment on the general theoretical connection between the perturbative and DS-based approaches: standardly in NLO, the symmetries of the medium (either gas, liquid, or solid, which are known from the specific point-group of the medium) are used in order to reduce the non-linear optical coefficients tensor to its minimal representation (to the smallest number of nonzero independent parameters). Using this approach, selection rules for specific perturbative NLO processes can be derived based on the elements of the reduced nonlinear optical tensor[10,11]. Our DS group theory based derivation generalizes this approach, giving rise to a unified description of selection rules for both perturbative NLO processes, and the non-perturbative processes (e.g., HHG).

Third, we discuss generalization of several selection rules by our approach:

(i) Generation of even-order harmonics is forbidden in centro-symmetric media[10,11]. This selection rule is re-derived for Hamiltonians exhibiting the DS $\hat{F}$. More generally, we find that the same selection rule is derived for Hamiltonians exhibiting the DS $\hat{C}_2$, such that even if the medium is not centro-symmetric and only exhibits $\hat{R}_2$ symmetry along the pump's propagation axis, the same selection rule is observed.
(ii) Rotationally-invariant crystals pumped by a monochromatic circularly polarized field generate only circularly polarized harmonics[14]. This selection rule is re-derived for Hamiltonians exhibiting the DS $\hat{C}_n$ (even when the pump field is not monochromatic). We also generalize this selection rule to the selection rules derived for Hamiltonians exhibiting the DSs $\hat{C}_{n,m}$, $\hat{e}_{n,m}$.
(iii) No HG in a spherically-symmetric media pumped by a monochromatic circularly polarized field[14]. This selection rule is re-derived for Hamiltonians exhibiting the DS $\hat{C}_n$ for $n \to \infty$. This means that this selection rule is also observed from media exhibiting a continuous rotational symmetry along the pump's propagation axis (cylindrical symmetry).

## Supplementary note 4: Examples of dynamical groups in (2+1)D

Examples of dynamical groups in (2+1)D are given in supplementary Table 1. In each group we specify the members of the group, and examples of vectorial functions which are characterized by the group.

**Supplementary Table 1 -** List of dynamical groups in (2+1)D, member DS operators, and example time-periodic vector functions that belong to each groups. A parametric Lissajou curve is given for each example field.

| ***Members*** | ***Order*** | ***Example function:*** $\mathbf{E}(t) = \begin{pmatrix} E_x \\ E_y \end{pmatrix}$ | ***Example Lissajou curve*** |
|---|---|---|---|
| $\{\hat{1}\}$ | 1 | $\begin{pmatrix} \cos(\omega t) + \cos(2\omega t) + \sin(3\omega t) \\ \sin(\omega t) - \sin(2\omega t) + \cos(3\omega t) \end{pmatrix}$ | 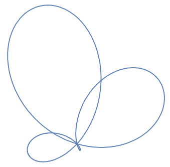 |
| $\left\{\hat{1}, \hat{C}_{n,m}, \dots, \left(\hat{C}_{n,m}\right)^{n-1}\right\}$ | $n$ | $\begin{pmatrix} \cos(\omega t) + \cos(2\omega t) + \sin(4\omega t) \\ \sin(\omega t) - \sin(2\omega t) - \cos(4\omega t) \end{pmatrix}$ | 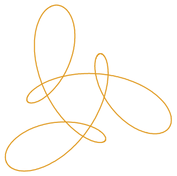 |
| $\left\{\hat{1}, \hat{e}_{n,m}, \dots, \left(\hat{e}_{n,m}\right)^{n-1}\right\}$ | $n$ | $\begin{pmatrix} \cos(\omega t) + \cos(2\omega t) + \sin(4\omega t) \\ b\sin(\omega t) - b\sin(2\omega t) - b\cos(4\omega t) \end{pmatrix}$ | 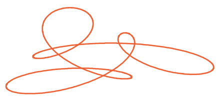 |
| $\{\hat{1}, \hat{T}\}$ | 2 | $\begin{pmatrix} \cos(3\omega t) \\ \cos(\omega t) + \cos(2\omega t) \end{pmatrix}$ | 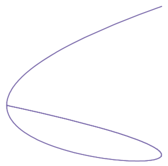 |
| $\{\hat{1}, \hat{S}\}$ | 2 | $\begin{pmatrix} \cos(\omega t) + \cos(2\omega t) \\ \sin(\omega t) + \sin(2\omega t) \end{pmatrix}$ | 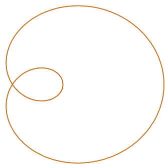 |
| $\{\hat{1}, \hat{Z}\}$ | 2 | $\begin{pmatrix} \cos(\omega t) \\ \sin(2\omega t + \pi/3) \end{pmatrix}$ | 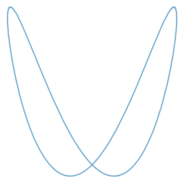 |
| $\{\hat{1}, \hat{H}\}$ | 2 | $\begin{pmatrix} \sin(\omega t) \\ \sin(2\omega t) + \cos(3\omega t)) \end{pmatrix}$ | 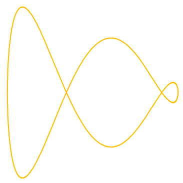 |
| $\{\hat{1}, \hat{Q}\}$ | 2 | $\begin{pmatrix} \sin(\omega t) \\ \sin(2\omega t) + \sin(3\omega t)) \end{pmatrix}$ | 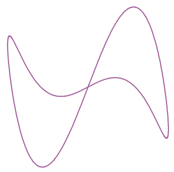 |
| $\{\hat{1}, \hat{G}\}$ | 2 | $\begin{pmatrix} \cos(\omega t) \\ \sin(2\omega t) + \cos(3\omega t)) \end{pmatrix}$ | 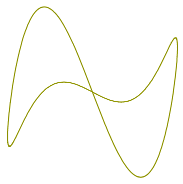 |

| | | | |
|---|---|---|---|
| $\{\hat{1}, \hat{D}_x, \hat{C}_2, \hat{H}_y\}$ | 4 | $\begin{pmatrix}\cos(\omega t) + \cos(3\omega t)\\ \sin(\omega t) + \sin(3\omega t)\end{pmatrix}$ | 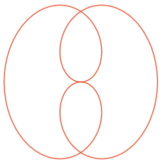 |
| $\{\hat{1}, \hat{H}_x, \hat{Q}, \hat{Z}_y\}$ | 4 | $\begin{pmatrix}\sin(\omega t)\\ \sin(2\omega t)\end{pmatrix}$ | 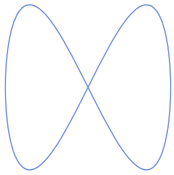 |
| $\{\hat{1}, \hat{Z}_x, \hat{G}, \hat{D}_y\}$ | 4 | $\begin{pmatrix}\cos(\omega t) - \cos(3\omega t)\\ \sin(2\omega t)\end{pmatrix}$ | 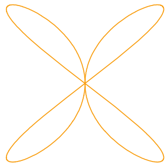 |
| $\{\hat{1}, \hat{C}_2, \hat{\mathrm{T}}, \hat{G}\}$ | 4 | $\begin{pmatrix}\cos(\omega t) + \cos(3\omega t)\\ \cos(3\omega t)\end{pmatrix}$ | 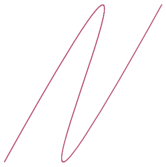 |
| $\{\hat{1}, \hat{Z}_x, \hat{\mathrm{T}}, \hat{H}_x\}$ | 4 | $\begin{pmatrix}\cos(2\omega t) + \cos(4\omega t)\\ \cos(\omega t)\end{pmatrix}$ | 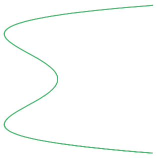 |
| $\left\{\hat{1}, \hat{C}_{n,m}, \dots, (\hat{C}_{n,m})^{n-1}, \hat{D}, \dots, \hat{D}(\hat{C}_{n,m})^{n-1}\right\}$ | $2n$ | $\begin{pmatrix}\cos(\omega t) + \cos(2\omega t)\\ \sin(\omega t) - \sin(2\omega t)\end{pmatrix}$ | 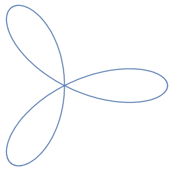 |
| $\left\{\hat{1}, \hat{e}_{n,m}, \dots, (\hat{e}_{n,m})^{n-1}, \hat{D}, \dots, \hat{D}(\hat{e}_{n,m})^{n-1}\right\}$ | $2n$ | $\begin{pmatrix}\cos(\omega t) + \cos(2\omega t)\\ b\sin(\omega t) - b\sin(2\omega t)\end{pmatrix}$ | 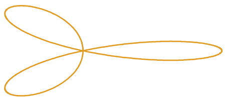 |

## Supplementary note 5: Similarities to other groups

As mentioned in the main text, dynamical Floquet groups are similar to those of line-groups[15] for the (2+1)D case. Considering line-groups that extend an infinite and periodic $z$-axis, this axis is similar to a periodic and infinite time-axis. Translations along the $z$-axis in line-groups are equivalent to time-translations ($\hat{\tau}_n$). All operations within the xy plane are identical to the spatial symmetry elements in (2+1)D dynamical groups. The main difference is in the time-reversal operation ($\hat{\mathrm{T}}$), which geometrically is similar to a spatial reflection plane within the $xy$ plane in line-groups. However, time-reversal is an anti-unitary operation unlike reflections. We further note that certain features of space-time groups[16], knot-groups[17], and space-groups[18], are isomorphic to Floquet groups. Due to the resemblence, we do not derive here the full list of character tables and multipcation tables of Floquet groups, but refer the reader to refs. [15–18], and references therein.

## Supplumentary note 6: TDSE calculations

### Numerical model

We numerically solve TDSE (2D/3D) in the length gauge, within the single active electron approximation, and the dipole approximation. In atomic units the TDSE is given by:

$$i\partial_t|\psi(t)\rangle = \left(-\frac{1}{2}\nabla^2 + V_{atom}(\mathbf{r}) + \mathbf{r}\cdot\mathbf{E}(t)\right)|\psi(t)\rangle \tag{32}$$

where $|\psi(t)\rangle$ is the time dependent wave function of the single electron and $V_{atom}(\mathbf{r})$ is the atomic potential. We use a spherical atomic model, where the electron is initially in the 1s orbital ground state found by complex time propagation. The Coulomb interaction is softened at the origin with two free parameters set to describe the ionization potential ($I_p$) of the Ne atom ($I_p = 0.793$ a.u.), and $He^+$ ion ($I_p = 1.999$ a.u.). We set the atomic potential according to ref. 19:

$$V_{atom}(\mathbf{r}) = -\frac{Z}{\sqrt{\mathbf{r}^2 + a}} \tag{33}$$

where $Z = 1, 2$, and $a = 0.1195, 0.1583$ a.u., for Ne and $He^+$ in the 2D case, respectively, and Z= $1.285$, a=0.0001 for Ne in the 3D case. To avoid reflections from the boundaries, we use a complex absorbing potential set to:

$$V_{ab}(\mathbf{r}) = -i\eta(|\mathbf{r}| - r_0)^{\alpha}\Theta(|\mathbf{r}| - r_0) \tag{34}$$

where $\eta = 5\times10^{-4}$, $\alpha = 3$, and $r_0 = 36$ a.u. in the 2D case, and $\eta = 7\times10^{-4}$, $\alpha = 4$, and $r_0 = 32$ a.u. in the 3D case, and $\Theta$ is a Heaviside step function. The peak intensity of the laser ($I_0$) is set in the range of $10^{13}$-$10^{14}$ W/cm$^2$ in all calculations, such that the overall ionization does not exceed 5%. The driving pulse has a flat-top envelope, where the rise and drop sections are five optical cycles long, and the flat top is five optical cycles long in the 2D case, and the rise and drop sections are four optical cycles long, and the flat top is six optical cycles long in the 3D case, unless stated otherwise.

In order to solve supplementary Eq. (32), we used the 3$^{rd}$ order split operator method[20,21]. The time and spatial grids were discretized on an $L\times L$ Cartesian grid for $L = 120$ a.u. in 2D, and an $L\times L\times L$ Cartesian grid for $L = 90$ in 3D, with spacing $dx = dy = dz = 0.2348$ a.u., and $dt = 0.02$ a.u.. Convergence was tested with respect to the grid densities, sizes, time step, and absorbing potential. The dipole acceleration was calculated using Ehrenfest theorem[22] as:

$$\mathbf{a}(t) = -\langle\psi(t)|\nabla V_{atom} + \mathbf{E}(t)|\psi(t)\rangle \tag{35}$$

The harmonic spectra was calculated as the Fourier transform of supplementary Eq. (35). Projections of the circular components of the dipole acceleration are calculated according to:

$$\tilde{a}_{+/-}(\Omega) = \tilde{a}_x(\Omega) \pm i\tilde{a}_y(\Omega) \tag{36}$$

where (+/-) refers to a positive/negative helicity, and "~" to the Fourier transformed component of the dipole.

### Numerical results

### $\hat{\boldsymbol{Z}}$ symmetry

We numerically show that the (2+1)D reflection-translation DS, $\hat{Z} = \hat{\tau}_2\cdot\hat{\sigma}$, results in the analytically derived selection rules. The results are presented in supplementary Fig. 1 for several exemplary cases. Each harmonic spectra shows the $x$ and $y$ components of the emitted harmonics with the numerically calculated ellipticity at each peak. The different drivers are presented in the inset of each harmonic spectra as a parametric Lissajous curve. The $xy$ components of the field are given up to an overall amplitude coefficient. The analytically derived selection rules for drivers with $\hat{Z}$ symmetry are such that all harmonics are linearly-polarized, where even and odd harmonics are oriented along, and perpendicular, to the symmetry axis, respectively. As seen in supplementary Fig. 1, the harmonic selection rules are generally upheld very well by all harmonic orders, yet deviations can appear near resonances, which are marked with dashed lines. For instance, as shown in supplementary Fig. 1(c), the ellipticity of the 10'th harmonic, which is near a resonance, is 0.09 instead of 0. As shown in supplementary Fig. 2, the deviation reduces when the length of the pulse or the length of the turn-on period of the flat-top envelope are increased. Thus, we conclude that resonances increase the sensitivity of

HHG to broken time translation symmetry (as exists in finite pulses) – an important and potentially useful feature in HHG spectroscopy through DS breaking.

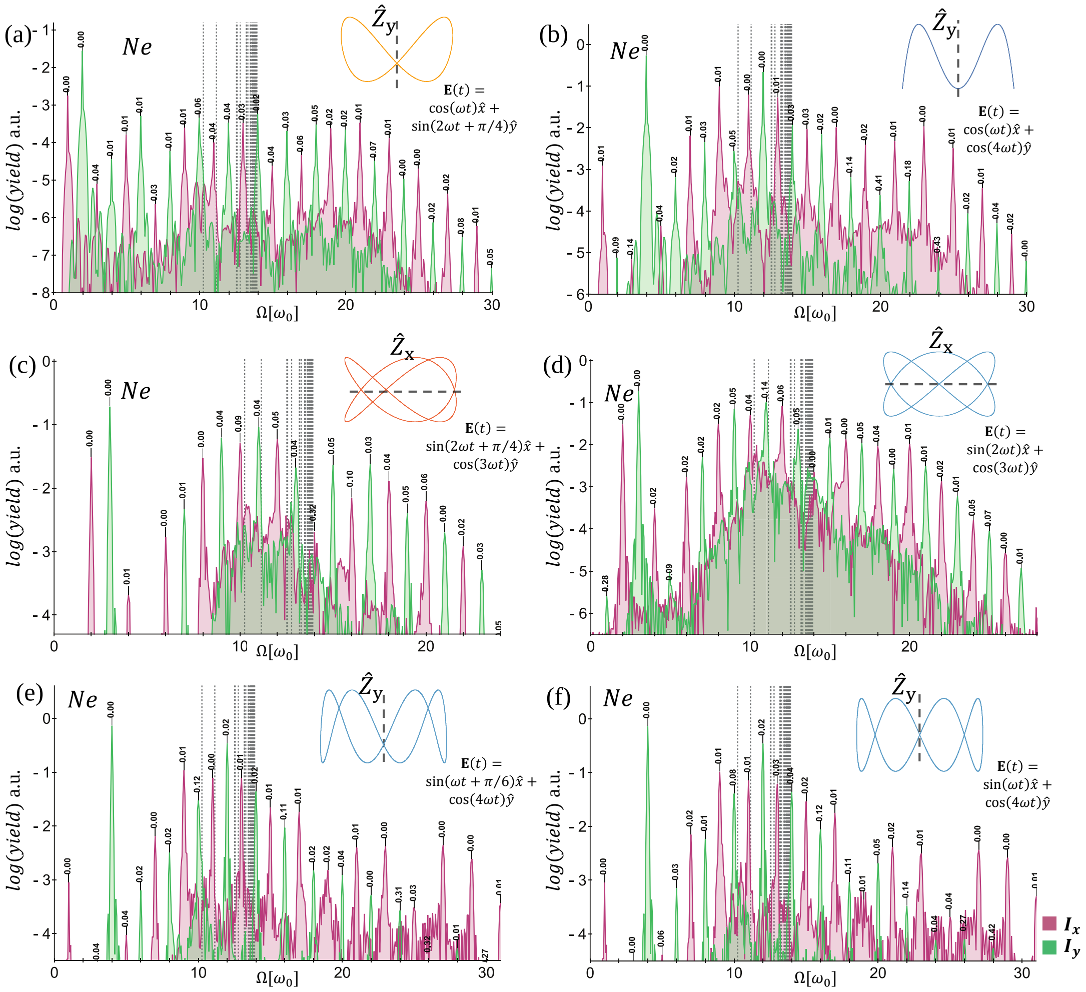


**Supplementary Fig. 1** HG spectra for driver fields with $\hat{\boldsymbol{Z}}$ DS from a model Ne atom system. **a-f** HG spectra from various types of driver fields that exhibit $\hat{\boldsymbol{Z}}$ DS. The driver field is indicated in inset up to an overall amplitude coefficient with its Lissajous curve. Atomic resonances are indicated by dashed lines, where the last line indicates the $I_p$. According to the selection rules derived from group theory, all harmonics should be linearly polarized, even harmonics along the symmetry axis, and odd harmonics orthogonal to it. The calculated ellipticity is presented at each peak, indicated in black, and intensity in the $y$-axis is given in log scale.

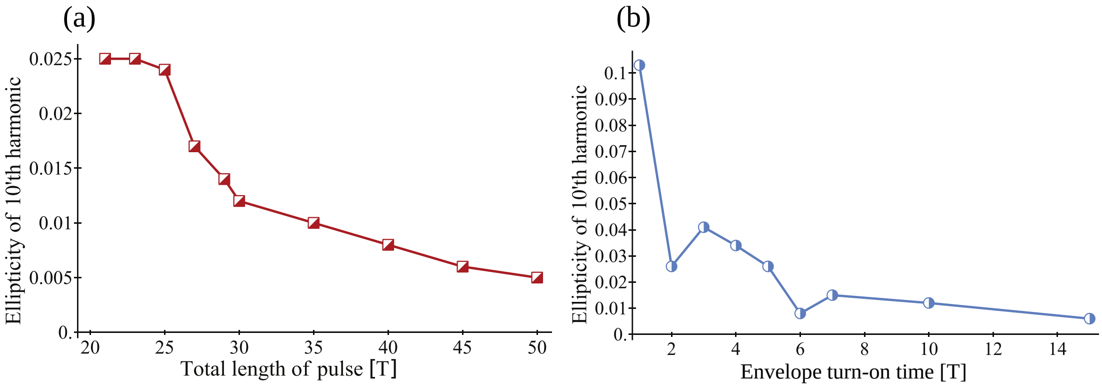


**Supplementary Fig 2** Time-translation symmetry breaking near atomic resonances. The ellipticity of the 10'th harmonic near an atomic resonance from supplementary Fig. 1(c) is shown as a function of changes in the pulse envelope that perturb time-translation symmetry. **a** Ellipticity as a function of the total pulse duration for an envelope with a rise and drop sections 10 optical cycles long, and a flat top of varying duration. **b** Ellipticity as a function of varying turn-on duration in the flat-top envelope, with a flat-top part that is 10 optical cycles long. With increasing pulse duration (and turn-on duration), the ellipticity indeed approaches zero, as predicted by the selection rules for the symmetry in this system.

**Time-reversal related symmetries ($\hat{Q}, \hat{G}, \hat{T}$)**

Next, we examine (2+1)D DS operators that involve time-reversal symmetries ($\hat{T}$ , $\hat{Q} = \hat{T} \cdot \hat{R}_2, \hat{G} = \hat{T} \cdot \hat{\tau}_2 \cdot \hat{R}_2$). According to group theory, these DSs should result in only linearly-polarized harmonics. However, these symmetries are slightly broken due to tunneling ionization (see main text). We numerically examine two systems with different ionization potentials: Ne and $He^+$. In $He^+$, the electron is tightly bound by the deep potential well, which results in a high $I_p$, and a large number of below $I_p$ harmonics that do not break time-reversal symmetry (since ionization does not play a role in their generation). Various examples are presented in supplementary Figs. 3 and 4. Each harmonic spectra shows the harmonic intensity and the numerically calculated ellipticity at each peak. The different drivers are presented in the inset of each harmonic spectra as a parametric Lissajous curve. The $x$-$y$ components of the field are given up to an overall amplitude coefficient. As shown in supplementary Figs. 3 and 4, the harmonic selection rules are generally upheld by harmonic orders below the $I_p$, where deviations are mostly found near resonances. Above $I_p$, the predicted selection rules are sometimes upheld and sometimes not. For example, in the spectrum of supplementary Fig. 3(b), several high harmonics have a relatively large ellipticity (up to 0.5) due to the broken symmetry. In contrast, in the spectrum of supplementary Fig. 4(b), all harmonics are practically linearly-polarized. This broken symmetry thus depends on several parameters, such as the shape and intensity of the driver and of the atomic potential.

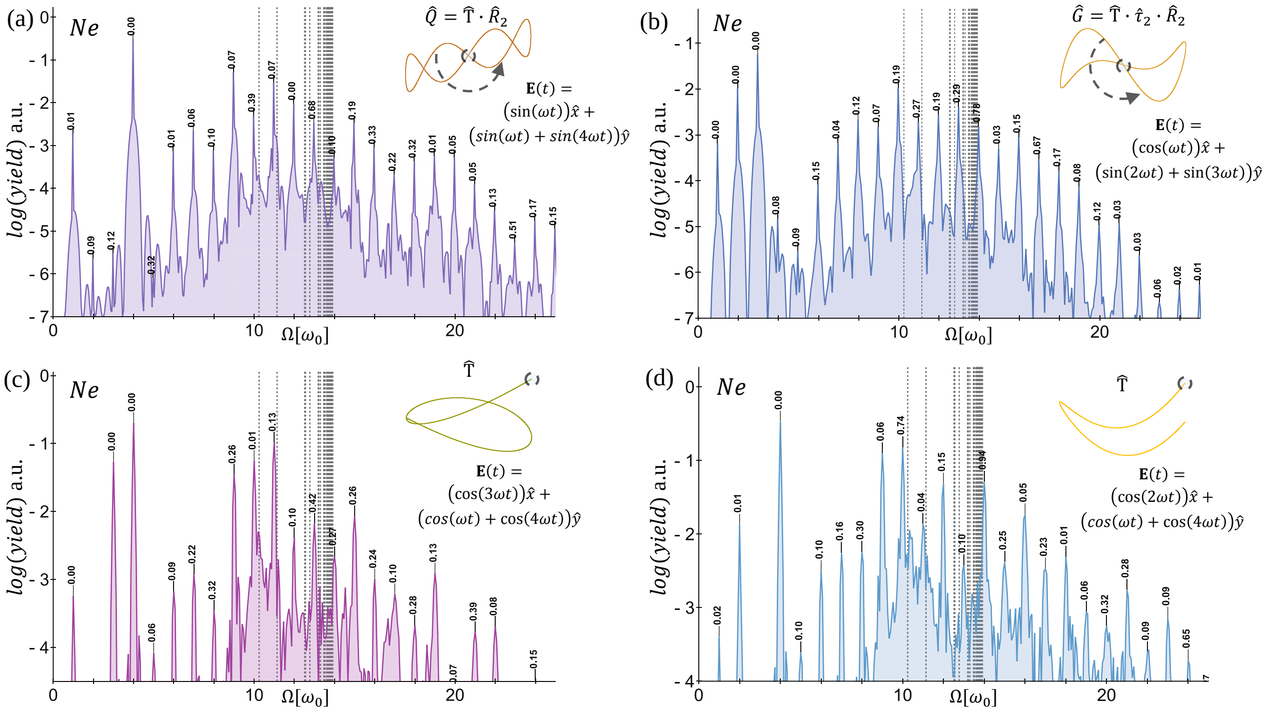


**Supplementary Fig. 3** HG spectrum for driver fields with DSs that involve time-reversal from a model Ne atom system. **a** a driving field with $\hat{Q}$ DS, **b** a driving field with $\hat{G}$ DS, **c** and **d** different driving fields with $\hat{T}$ DS. The DS is indicated in the inset of each spectrum with the driver field up to an overall amplitude coefficient, and its Lissajous curve. Atomic resonances are indicated by dashed lines, where the last line indicates the $I_p$. According to the selection rules derived from group theory all harmonics should be linearly-polarized. The calculated ellipticity is presented at each peak, indicated in black, and intensity in the y-axis is given in log scale.

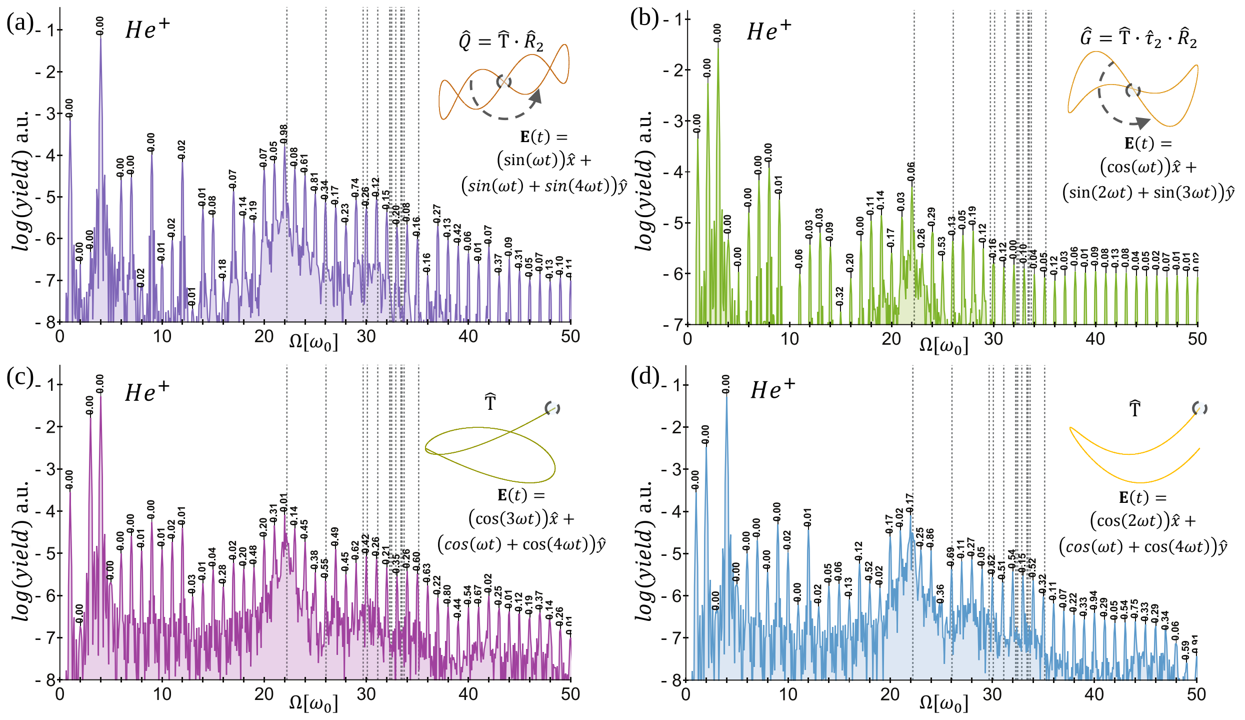


**Supplementary Fig. 4** HG spectrum for driver fields with DSs involving time-reversal from a model $He^+$ ion system. **a** a driving field with $\hat{\boldsymbol{Q}}$ DS, **b** a driving field with $\hat{\boldsymbol{G}}$ DS, **c** and **d** different driving fields with $\hat{\mathbf{T}}$ DS. The DS is indicated in inset of each spectrum with the driver field up to an overall amplitude coefficient, and its Lissajous curve. Atomic resonances are indicated by dashed lines, where the last line indicates the $I_p$. According to the selection rules derived from group theory all harmonics should be linearly-polarized. The calculated ellipticity is presented at each peak, indicated in black, and intensity in the y-axis is given in log scale.

## 4th order elliptical symmetry ($\hat{e}_4$)

The kinetic energy and atomic/molecular potential terms are generally not invariant under the elliptical symmetry operation, $\hat{e}_n$. Still, there are cases where a driver pump can be chosen to uphold an elliptical symmetry, and not break the spherical symmetry of other terms in the Hamiltonian. This can only happen for n=4, since every ellipse has four points that are equidistant from the origin (supplementary Fig. 5). For example, in ref. 23 a train of linearly-polarized pulses was proposed that upholds this symmetry relation, and it was indeed shown numerically to yield the predicted selection rules. In this case, within each pulse the electron's motion, and therefore the polarization, is confined to the polarization axis of the pulse. Thus, the polarization is invariant under the elliptical DS. Below, we present an intriguing case that also yields the predicted selection rules of the (2+1)D elliptical DS, even though the electron's motion is two dimensional and intricate. We explore HHG driven by a synthetic field defined by a piecewise function that upholds 4'th order elliptical symmetry. The driver field has the form:

$$\mathbf{E}(t) = \frac{E_0}{2} A(t)\, sin(2\omega_0 t) \times \begin{Bmatrix} \hat{x} + b\hat{y}; & 0 < t < T/4 \\ -\hat{x} + b\hat{y}; & T/4 < t < T/2 \\ -\hat{x} - b\hat{y}; & T/2 < t < 3T/4 \\ \hat{x} - b\hat{y}; & 3T/4 < t < T \end{Bmatrix} \tag{37}$$

where $E_0$ is the field amplitude, $A(t)$ is a flat-top envelope with a three optical cycle ramp up and down and a 15 optical cycle long flat-top, $b$ is the ellipticity of the underlying symmetry (from 0 to 1), and $\omega_0 = 2\pi/T$ is the optical frequency. Notably, producing this field requires an infinitely broad spectrum due to the non-smooth orientation shifts (the field is continuous but its derivative is not). Still, while such a field is not accessible experimentally, it can be approached by using multi-color drivers. This field provides 4th order elliptical symmetry present in all terms in the Hamiltonian. This example is slightly more general than the case of linearly-polarized pump trains, since the function in supplementary Eq. (37) maintains the symmetry on a sub-cycle level, where the electron dynamics is two dimensional and intricate. Numerical calculations of the HG spectra for several values of target ellipticity are shown in supplementary Fig. 6. Each peak shows the numerically

calculated ellipticity, and the (+) and (-) projected helical components to indicate the helicity of the harmonics. The Lissajou parametric curve in each case is shown in inset, along with the ellipticity of the driver's underlying symmetry. In all cases the selection rules derived from group theory match with the calculated spectrum, except near atomic resonances.

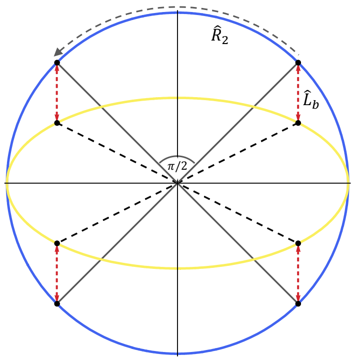


**Supplumentary Fig. 5** Schematic illustration of 4th order elliptical DS ($\hat{\boldsymbol{e}}_4$) which still maintains circular DS for any ellipticity. In every ellipse four points equidistant from the origin exist, which belong both to a circle and an ellipse simultaneously. The red arrows represent scaling transformation for the $y$ axis from the blue unit circle to the yellow ellipse.

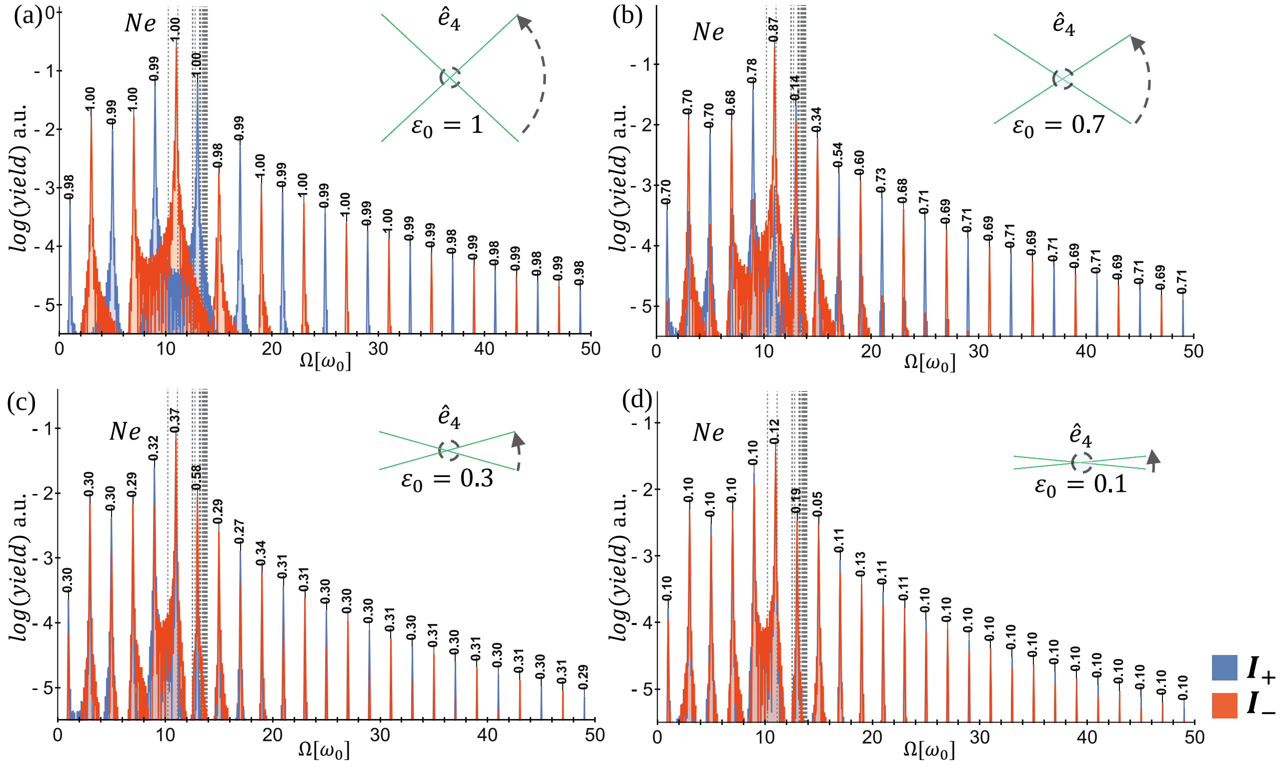


**Supplementary Fig. 6** HG spectrum from a 4th order elliptical DS piecewise electric driver field, as in supplementary Eq. (37). **a-d**, underlying ellipticity ε0=1,0.7,0.3,0.1, respectively. The ellipticity of the underlying symmetry is indicated in inset, as well as the field Lissajous curve. Calculated ellipticity for each harmonic peak is indicated in black, and according to the selection rules derived from group theory should be identical to the ellipticity of the driver's symmetry, with alternating helicities. Atomic resonances are indicated in dashed lines, where the last line indicates the $I_p$. Intensity in the y-axis is projected onto the left and right circularly polarized components, in red and blue, respectively, and the intensity is given in log scale.

### (3+1)D Rotational symmetry in non-collinear HG ($\hat{\boldsymbol{C}}_{n,m}$)

Next, we examine (3+1)D rotational DS operators ($\hat{C}_{n,m} = \hat{\tau}_n \cdot \hat{R}_{n,m}$). These differ from the (2+1)D case by a specific choice of rotational symmetry axis, which we denote the $z$-axis. According to group theory, this DS should result in counter rotating $nq \pm m$ harmonics that are circularly polarized within the $xy$ plane, and also $nq$ harmonics that can only be emitted with linear polarization along the $z$-axis (for integer $q$). Fascinatingly, emission of the $nq$ harmonics means that there is photon mixing between photons with all possible polarization axes (since otherwise the even $nq$ harmonics would be forbidden). We numerically verify this in a Ne like 3D

system for a non-collinear geometry with $\hat{C}_3$ symmetry, where HG is driven by a counter rotating $\omega$-$2\omega$ bi-circular pump propagating along the $z$-axis, and an orthogonally propagating (along the x-axis) pulse of frequency $3\omega$ polarized linearly along the $z$-axis, seen in supplementary Fig. 7. The spectrum shows the harmonic intensity projected onto the circular components within the $xy$ plane with the numerically calculated ellipticity at each peak, as well as the remaining $z$-axis emission. The driving field is presented in the inset as a parametric Lissajous curve, and schematic illustration of the non-collinear setup.

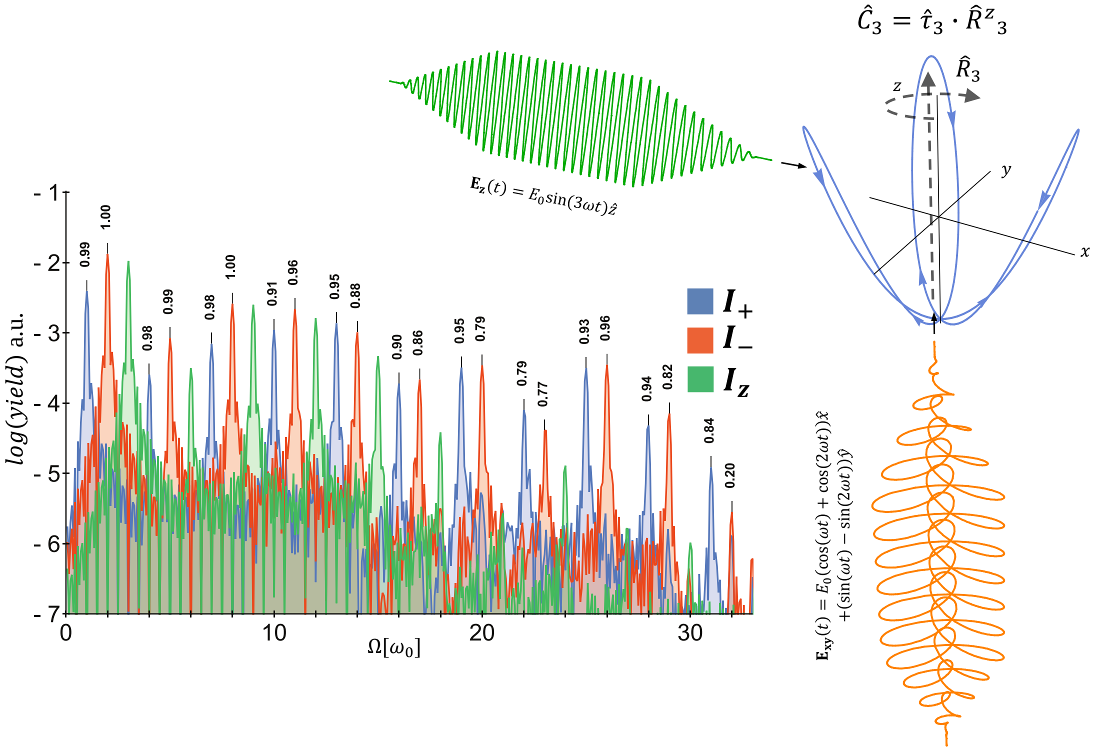


**Supplementary Fig. 7** HG spectrum from a (3+1)D $\hat{\boldsymbol{C}}_3$ symmetric pump. The driving field is given in the inset up to an over amplitude coefficient. Red and blue stand for the circular projection within the *xy* plane, and calculated ellipticity for each harmonic peak within is indicated in black, and according to the selection rules should be exactly 1, with alternating helicities. The z-axis emission is presented in green, and is shown to be only at the predicted harmonic frequencies $3q$ (for integer $q$). Intensity is given in log scale.

### (3+1)D Improper Rotational symmetry in noncollinear HG ($\widehat{\boldsymbol{M}}_{n,m}$)

Next, we examine (3+1)D rotational DS operators ($\widehat{M}_{2n,m} = \hat{\tau}_{2n} \cdot \hat{R}_{2n,m} \cdot \hat{\sigma}_h$). According to the presented theory, this DS results in counter rotating $2nq \pm m$ harmonics that are circularly polarized within the xy plane, and also $n(2q+1)$ harmonics that can only be emitted with linear polarization along the $z$-axis (for integer $q$). The emission of the $n(2q)$ harmonics is symmetry forbidden, which is very surprising because this gives the appearance that selection rules from each axis are upheld separately, as if no mixing occurs. As an example, we explore $\widehat{M}_{4,1}$ symmetry in a non-collinear geometry, with a bi-circular $\omega$-$3\omega$ counter-rotating pump pulse propagating along the $z$-axis, and an orthogonally propagating pump pulse (propagating along the x-axis) that is linearly polarized along the $z$-axis at frequency $2\omega$. In this case, harmonics 3,5,7,9,11… should be circularly polarized with alternating helicities within the $xy$ plane. Harmonics 2,6,10,14,18… should be emitted only with polarization components along the $z$-axis. The remaining 4,8,12,16… harmonics are symmetry forbidden. We numerically verify this in a Ne like system, seen in supplementary Fig. 8. The spectrum shows the harmonic intensity projected onto the circular components within the $xy$ plane with the numerically calculated ellipticity at each peak, as well as the remaining $z$-axis emission. The driving field is presented in the inset as a parametric Lissajou curve, and schematic illustration of the non-collinear setup.

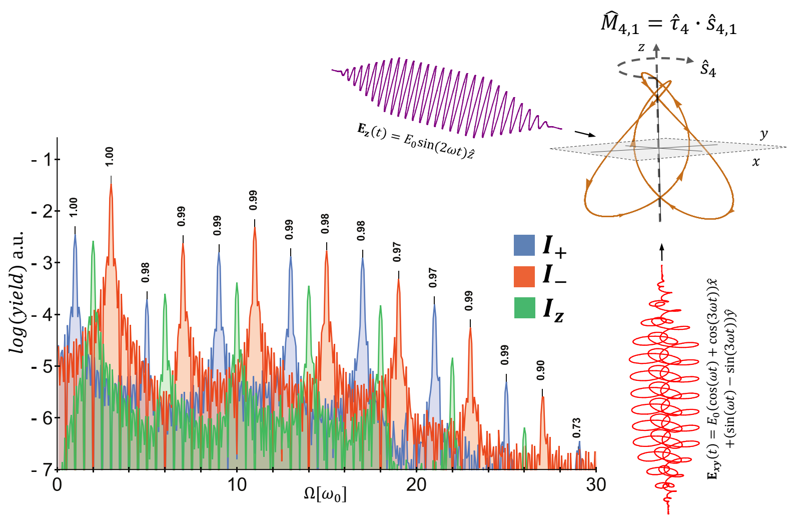


**Supplementary Fig. 8** HG spectrum from a (3+1)D $\hat{\boldsymbol{M}}_{\mathbf{4,1}}$ symmetric pump. The driving field is given in the inset up to an over amplitude coefficient. Red and blue stand for the circular projection within the *xy* plane, and calculated ellipticity for each harmonic peak within is indicated in black, and according to the selection rules should be exactly 1, with alternating helicities. The z-axis emission is presented in green, and is shown to be only at the predicted harmonic frequencies 2(2*q*+1) for integer *q*. Intensity is given in log scale.

## Supplementary note 7: HG selection rules from molecules and DS breaking spectroscopy

Here we present numerical examples for molecular HG selection rules, and for molecular symmetry and orientation spectroscopy from DS breaking. Specifically, we consider a planar 3-fold symmetric molecule that belongs to the $D_{3h}$ point group (which supports $120^0$ rotations, $180^0$ rotations, and also reflections along the major molecular axes, within the BO approximation), interacting with a pump field that exhibits $\hat{Z} = \hat{\tau}_2\hat{\sigma}$ DS. We demonstrate that the selection rules derived from Floquet group theory depend both on the pump's DS, and on the symmetries of the molecule when it is oriented, and that the relative alignment with respect to the pump field determines whether a selection rule is observed (which is used for orientation spectroscopy). In case the medium is non-oriented, we show that the symmetry group of the orientation averaged ensemble and the pump's DS determine the observed selection rules.

According to the Floquet group theory, we should observe $\hat{Z}$ DS based selection rules in the HG spectrum from oriented molecules if the reflection axis of the pump is co-aligned with that of the molecules, leading to linearly polarized harmonics, where even/odd harmonics are polarized parallel/perpendicular to the symmetry axis. When the axes are not aligned, symmetry breaking will occur due to the molecular potential, and the selection rule will be broken. For the random non-oriented medium, theory predicts that the selection rule will be observed, because the orientation averaged ensemble is *O(3)* spherically symmetric (as discussed in the main text, and will only differ for chiral media), and supports the required reflection symmetry.

We consider the following 2D molecular potential that describes a poly-atomic molecule:

$$V_{mol}(x,y) = -\sum_{i=1}^{3} \frac{1/3}{\sqrt{(x-x_i)^2 + (y-y_i)^2}} \tag{38}$$

where we set $(x_i, y_i) = R_0(\cos(\alpha_i + \theta), \sin(\alpha_i + \theta))$, for $R_0 = 2.5$ a.u., $\alpha_i = \{0, 120^0, 240^0\}$, i=1,2,3, and we set $\theta$ as the relative angle of the molecule with respect to the $x$-axis. This potential is represented on a real-space Cartesian $L \times L$ grid for $L = 120$ a.u., with grid spacing $\Delta x = \Delta y = 0.1171$ a.u., where the ground state and first excited states are found by complex time propagation. The TDSE is solved as in supplementary note 6 for the case where the molecule is interacting with the following cross-linear $\omega$-$2\omega$ driving field that possesses the reflectional $\hat{Z}$ DS, but not 3-fold rotational DSs:

$$\mathbf{E}(t) = E_0 A(t)(\sin(\omega_0 t)\,\hat{x} + \sin(2\omega_0 t)\,\hat{y}) \tag{39}$$

where $A(t)$ is a flat-top envelope with a 4 optical cycle ramp up and down and an 6 optical cycle long flat-top, $\omega_0 = 2\pi/T$ is the optical frequency that corresponds to an 800nm wavelength, and $E_0$ is chosen such that the maximal intensity of the pulse is $10^{13}$[W/cm$^2$]. The TDSE is solved for many relative orientations with respect to the laser field, such that we obtain the HG spectrum as a function of $\theta$ and the HG spectrum from the random non-oriented media (see supplementary Fig. 9).

Supplementary Fig. 9(a) shows that when the molecular axis is aligned with the pump's reflection axis, the selection rules for $\hat{Z}$ symmetry are observed. On the other hand, these selection rules are broken if the axes are not aligned (supplementary Fig. 9(b)). Furthermore, for the non-oriented medium, the selection rules from the pump's symmetry are upheld regardless of the molecular medium (supplementary Fig. 9(c)). In fact, most HG experiments deal with such non-oriented media, meaning usually the observed selection rules are identical to those observed from atomic media[24] (this is why the majority of numerical examples above are presented for the atomic case). We also note that the molecule's symmetry properties that are not exhibited by the pump field are washed out in the spectrum, that is, there is no selection rule due to 3-fold symmetry even if the medium is aligned. This is because a symmetry based selection rule is only observed if the full Hamiltonian supports the DS, and in this case the pump is not 3-fold symmetric. Importantly, similar results are obtained when the TDSE is solved with other initial states (and also from degenerate states, as long as an equal population of degenerate states is chosen), and in a full 3D molecular potential. All of these results match the predictions by the Floquet group theory.

Next, we consider DS breaking spectroscopy for the molecule with $\hat{Z}$ symmetry - we can identify the molecular orientation in aligned media by examining when the selection rule from the pump is restored (orientation spectroscopy was demonstrated experimentally in $SF_6$ molecular media through a rotational DS in ref. 24). This is shown in supplementary Fig. 10(a) for the average intensity of the even/odd harmonic spectrum polarized along the $x/y$ axes, respectively, and in supplementary Fig. 10(b) for the average ellipticity of the spectrum (harmonics 3-15). As seen, when the molecule is aligned with the pump, the selection rule is restored (hence $\theta$ can be measured), and we can also identify the presence of three reflection planes in the molecule. Conceptually, one can employ multiple pump fields of varying DSs and deduce the full molecular point group in this manner.

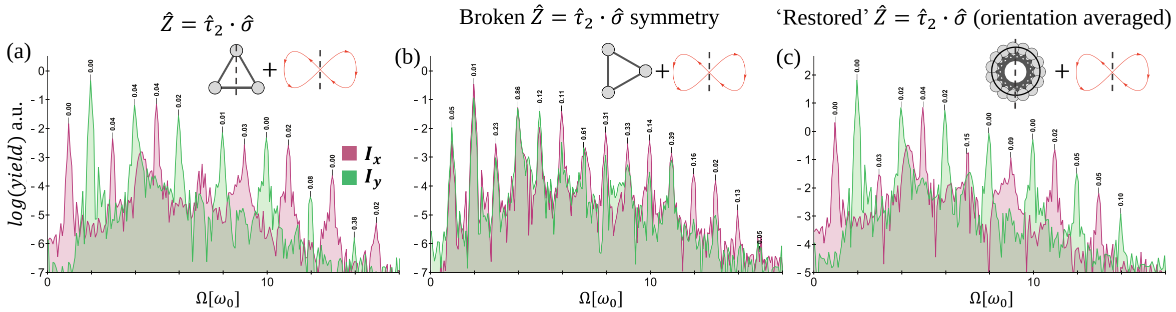


**Supplementary Fig. 9** Molecular HG spectrum from a $\hat{\boldsymbol{Z}}$ symmetric pump and 3-fold molecular potential. The driving field's Lissajou plot is given in the inset together with the molecular orientation. The calculated ellipticity for each harmonic peak is indicated in black. Intensity is given in log scale. **a** Symmetric case, **b** symmetry broken case, **c** orientation averaged spectra (symmetric case).

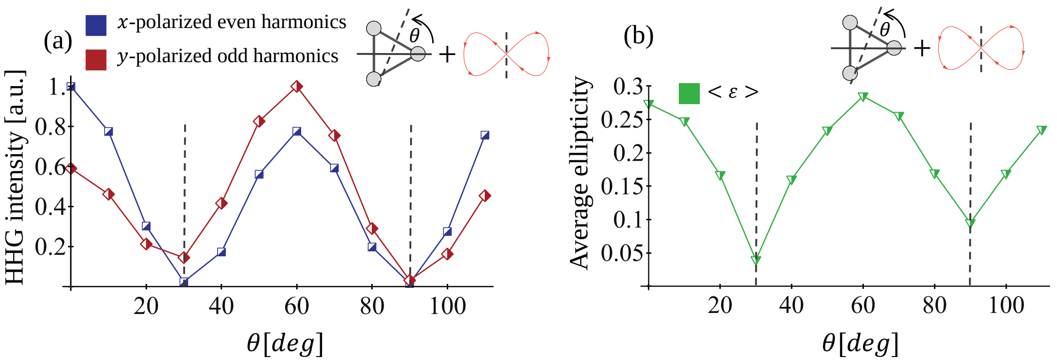


**Supplementary Fig. 10** Molecular HG DS breaking orientation spectroscopy from a $\hat{\boldsymbol{Z}}$ symmetric pump and a 3-fold molecular potential. The driving field's Lissajou plot is given in the inset together with the molecule's orientation. **a** Intensity spectroscopy – the selection rules from the pump field dictate that *x*-polarized harmonics are only odd, and *y*-polarized harmonics are only even, which is upheld when the molecule's reflection axis is aligned with the pump. **b** Ellipticity spectroscopy – the selection rules from the pump field dictate that the spectrum is fully linearly polarized (0 ellipticity), which is upheld when the molecular axis is aligned with the pump.

## Supplementray note 8: HG selection rules from solids and DS breaking orientation spectroscopy

Here we give some examples for new selection rules for HG from solids. In this particular case, the symmetries of the solid and the respective angle of incidence of the pump can determine the symmetries of the system. For example, a monochromatic laser field of any ellipticity upholds the DS $\hat{F} = \hat{\tau}_2 \cdot \hat{\imath}$. If the solid has an inversion center (centro-symmetry) $H_{HG}$ is invariant, and generation of even harmonics become forbidden. In fact, this is a well-known symmetry based selection rule in nonlinear optics[10,14] that is usually attributed to the symmetry of the solid, and our derivation presents its generalization. A second noteworthy example is that of the DS $\hat{Z} = \hat{\tau}_2 \cdot \hat{\sigma}$, which is a symmetry of any monochromatic linearly polarized laser field. When driving a solid with a reflection symmetry plane that is aligned orthogonal to the pump's polarization axis $H_{HG}$ is again invariant, leading to orthogonally linearly polarized even and odd harmonics. Importantly, if the solid is tilted out of plane with the incident wave, symmetry breaking occurs, which allows spectroscopically finding the solid's orientation. Lastly, DSs could be used to probe spin-orbit coupling or magnetic interactions in the solid, since these terms could break certain DSs.

As a numerical example, we show that the solid's orientation can break the reflection DS $\hat{Z}$ if it is not aligned to the pump's orientation, and use this to demonstrate orientation spectroscopy in solid HG. We calculate the harmonic spectrum from a single electron in a model 2D Mathieu type lattice potential[25]

$$V_{solid}(x,y) = -V_0\left(1+\cos\left(\frac{2\pi}{a}x\right)\right)\left(1+\cos\left(\frac{2\pi}{a}y\right)\right) \tag{40}$$

where $V_0 = 0.25$, $a = 10$ a.u. This is done similarly to the atomic systems, on a real-space Cartesian grid spanning $20 \times 20$ lattice sites of size $L \times L$ for $L = 370$ a.u., with grid spacing $\Delta x = \Delta y = 0.2645$ a.u. The ground state is found by complex time propagation that corresponds to a real-space delocalized wave function that populates the first valence band, as is developed in ref. 26. The TDSE is then solved as in supplementary note 6 with the following cross-linear $\omega$-$2\omega$ driving field that possesses only $\hat{Z}$ DS:

$$\mathbf{E}(t) = E_0 A(t)(\sin(\omega_0 t + \phi)\,\hat{x} + \sin(2\omega_0 t)\,\hat{y}) \tag{41}$$

where $\phi = \pi/3$, $A(t)$ is a flat-top envelope with a 5 optical cycle ramp up and down and an 8 optical cycle long flat-top, $\omega_0 = 2\pi/T$ is the optical frequency that corresponds to an 800nm wavelength, and $E_0$ is chosen such that the maximal intensity of the pulse is $5 \times 10^{13}$[W/cm$^2$]. The harmonic spectrum is calculated and seen in supplementary Fig. 11 projected onto $x$ and $y$ polarization components for two cases: (a) the solid is aligned with the pump and the two share a $y$-axis reflection DS, and (b) the solid is rotated by $25^0$ with respect to the DS axis (this Mathieu potential supports three reflection axes). From supplementary Fig. 11, clearly when the DS is upheld the spectrum contains orthogonally polarized even and odd harmonics only, but for the broken symmetry different polarizations are emitted for different harmonics. We use this for orientation spectroscopy in supplementary Fig. 11(c), which shows the calculated average ellipticity of harmonics 10-18 in the spectrum as a function of the solid's angle with respect to the pump field. The average ellipticity has strong minima at

$\alpha = 0^0, 45^0, 90^0$, which are the exact angles of reflection planes in the solid. Hence, an experimental ellipticity measurement may deduce the solid's orientation through DS breaking spectroscopy, and also the existence or lack of reflection planes in the solid. Notably we also see a peak in the average ellipticity at $\alpha \approx 25^0$, which is the orientation farthest away from any reflection axis, meaning that the maximal symmetry breaking may also be a useful observable.

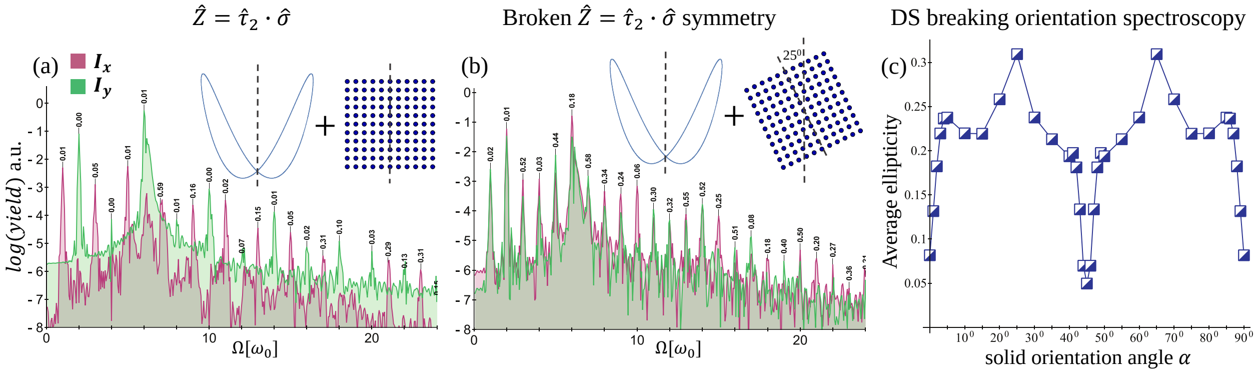


**Supplementary Fig. 11** Solid HG spectrum from a $\hat{\boldsymbol{Z}}$ symmetric pump and a Mathieu type potential used for orientation spectroscopy. The driving field's Lissajou plot is given in the inset together with the solid's orientation. The calculated ellipticity for each harmonic peak is indicated in black. Intensity is given in log scale. **a** Symmetric case, **b** symmetry broken case, **c** average ellipticity of harmonics 10-18 in the spectrum with respect to the solid's orientation, where deep minima indicate reflection planes in the solid, and can be used to find the solid's orientation.

## Supplumentary references


1. Kfir, O. *et al.* Generation of bright phase-matched circularly-polarized extreme ultraviolet high harmonics. *Nat. Photonics* **9,** 99–105 (2014).
2. Dudovich, N. *et al.* Measuring and controlling the birth of attosecond XUV pulses. *Nat Phys* **2,** 781–786 (2006).
3. Shafir, D., Mairesse, Y., Villeneuve, D. M., Corkum, P. B. & Dudovich, N. Atomic wavefunctions probed through strong-field light–matter interaction. *Nat. Phys.* **5,** 412–416 (2009).
4. Ben-Tal, N., Moiseyev, N. & Beswick, A. The effect of Hamiltonian symmetry on generation of odd and even harmonics. *J. Phys. B At. Mol. Opt. Phys.* **26,** 3017 (1993).
5. Weihe, F. A. *et al.* Polarization of high-intensity high-harmonic generation. *Phys. Rev. A* **51,** R3433–R3436 (1995).
6. Antoine, P., Carré, B., L'Huillier, A. & Lewenstein, M. Polarization of high-order harmonics. *Phys. Rev. A* **55,** 1314–1324 (1997).
7. Alon, O. E., Averbukh, V. & Moiseyev, N. Selection Rules for the High Harmonic Generation Spectra. *Phys. Rev. Lett.* **80,** 3743–3746 (1998).
8. Milošević, D. B. Circularly polarized high harmonics generated by a bicircular field from inert atomic gases in the p state: A tool for exploring chirality-sensitive processes. *Phys. Rev. A* **92,** 043827 (2015).
9. Fan, T. *et al.* Bright circularly polarized soft X-ray high harmonics for X-ray magnetic circular dichroism. *Proc. Natl. Acad. Sci. U. S. A.* **112,** 14206–14211 (2015).
10. Yariv, A. & Yeh, P. *Photonics: optical electronics in modern communications*. (Oxford University Press, 2007).
11. Boyd, R. W. *Nonlinear optics*. (Elsevier, 2003).
12. Fleischer, A., Kfir, O., Diskin, T., Sidorenko, P. & Cohen, O. Spin angular momentum and tunable polarization in high-harmonic generation. *Nat. Photonics* **8,** 543–549 (2014).
13. Ching, W. Y. & Xu, Y.-N. First-Principles Calculation of Electronic, Optical, and Structural Properties of α-Al2O3. *J. Am. Ceram. Soc.* **77,** 404–411 (1994).
14. Tang, C. L. & Rabin, H. Selection Rules for Circularly Polarized Waves in Nonlinear Optics. *Phys. Rev. B* **3,** 4025–4034 (1971).
15. Damnjanovic, M. & Miloševic, I. *Line Groups in Physics: Theory and Applications to Nanotubes and Polymers (Lecture Notes in Physics vol 801)*. (Berlin: Springer, 2010).
16. Janssen, T., Janner, A. & Ascher, E. Crystallographic groups in space and time. *Physica* **41,** 541–565 (1969).
17. Neuwirth, L. P. *Knot Groups. Annals of Mathematics Studies.* **56,** (Princeton University Press, 1965).

18. Hammond, C. *Basics of crystallography and diffraction*. (Oxford University Press, 2015).
19. Medišauskas, L., Wragg, J., Van Der Hart, H. & Ivanov, M. Y. Generating Isolated Elliptically Polarized Attosecond Pulses Using Bichromatic Counterrotating Circularly Polarized Laser Fields. *Phys. Rev. Lett.* **115,** 153001 (2015).
20. Fleck, J. A., Morris, J. R. & Feit, M. D. Time-dependent propagation of high energy laser beams through the atmosphere. *Appl. Phys.* **10,** 129–160 (1976).
21. Feit, M. D., Fleck, J. A. & Steiger, A. Solution of the Schrödinger equation by a spectral method. *J. Comput. Phys.* **47,** 412–433 (1982).
22. Burnett, K., Reed, V. C., Cooper, J. & Knight, P. L. Calculation of the background emitted during high-harmonic generation. *Phys. Rev. A* **45,** 3347–3349 (1992).
23. Neufeld, O., Bordo, E., Fleischer, A. & Cohen, O. High Harmonics with Controllable Polarization by a Burst of Linearly-Polarized Driver Pulses. *Photonics* **4,** 31 (2017).
24. Baykusheva, D., Ahsan, M. S., Lin, N. & Wörner, H. J. Bicircular High-Harmonic Spectroscopy Reveals Dynamical Symmetries of Atoms and Molecules. *Phys. Rev. Lett.* **116,** 123001 (2016).
25. Slater, J. C. A soluble problem in energy bands. *Phys. Rev.* **87,** 807 (1952).
26. Liu, X. *et al.* Wavelength scaling of the cutoff energy in the solid high harmonic generation. *Opt. Express* **25,** 29216–29224 (2017).
